# Laboratory and Microbiological Considerations in Sepsis-Induced Cardiac Dysfunction

**DOI:** 10.3390/medicina61101765

**Published:** 2025-09-30

**Authors:** Catalina Paraschiv, Denisa Oana Nicolaescu, Mihaela Roxana Popescu, Carmen Cristina Vasile, Emanuel Moisa, Silvius Ioan Negoita, Serban Mihai Balanescu

**Affiliations:** 1 Department of Cardiothoracic Pathology, University of Medicine and Pharmacy “Carol Davila”, 011461 Bucharest, Romania; catalina.paraschiv@drd.umfcd.ro (C.P.); serban.balanescu@umfcd.ro (S.M.B.); 2 Cardiology Unit, Elias Emergency University Hospital, University of Medicine and Pharmacy “Carol Davila”, 011461 Bucharest, Romania; 3 Department of Cardiovascular Diseases, Mayo Clinic, Rochester, MN 55905, USA; 4 Department of Epidemiology, National Institute for Infectious Diseases “Prof. Dr. Matei Bals”, University of Medicine and Pharmacy “Carol Davila”, 021105 Bucharest, Romania; 5 Department of Anaesthesia and Intensive Care Medicine, Elias Emergency University Hospital, University of Medicine and Pharmacy “Carol Davila”, 011461 Bucharest, Romania; emanuel.moisa@umfcd.ro (E.M.);

**Keywords:** sepsis, sepsis-induced cardiac dysfunction, echocardiography, cultures

## Abstract

*Introduction*: Sepsis-induced cardiac dysfunction (SICD) is a transient cardiac disfunction, with variable described prevalence and uncertain prognostic. This study aimed to characterize the laboratory and microbiological findings in critically ill patients with sepsis who developed left ventricular (LV) or biventricular systolic dysfunction. *Methods*: Patients who required intensive care unit hospitalization for sepsis were screened retrospectively. Only patients with positive cultures and echocardiography performed within 24 h from admission were included. The exclusion criteria were infective endocarditis, acute coronary syndrome, history of cardiomyopathy, severe valve disease, end-stage organ or oncological disease. Cardiac function was appreciated on transthoracic echocardiography, using LV ejection fraction for the left ventricle and tricuspid annular plane systolic excursion (TAPSE) for the right ventricle. SICD was confirmed if the systolic dysfunction found upon admission was reversible within 7–10 days. *Results*: A total of 100 patients with positive cultures were included. The median age was 73 and 55% were male. SICD was diagnosed in 14% of patients. Patients with SICD were more likely to develop septic shock and had longer hospital and intensive care unit stay. In-hospital mortality was 44% with no significant difference between SICD and non-SICD patients. Laboratory markers upon hospital admission showed that SICD patients had significantly higher values of lactate and transaminases. Cardiac (troponin and NT-proBNP) and inflammation markers (leukocytes, neutrophils, NLR, C-reactive protein, procalcitonin) had higher values in patients with SICD but the difference did not reach statistical significance. Streptococcal infections and polymicrobial cultures were risk factors for developing SICD. Higher rates of infections with *Enterobacterales* were seen in the SICD group but the difference was not significant. *Conclusions*: SICD patients had higher lactate, inflammation, and cardiac biomarkers levels upon admission and significantly higher rates of streptococcal infections and polymicrobial cultures.

## 1. Introduction

Sepsis is a severe, life-threatening organ dysfunction triggered by an anomalous response to a bacterial, fungal, viral or parasitic infection [1]. Despite recent advances in hospital care and medication, sepsis remains a clinical condition with significant rates of mortality and morbidity worldwide. It is estimated that 19.7% of all deaths worldwide are due to sepsis [2]. Mortality occurs in more than one-third of patients hospitalized for sepsis and is greater when patients require intensive care unit (ICU) stays [3]. The most common origin of infection leading to sepsis is the respiratory tract, followed by the genitourinary tract, abdominal, and wound and soft tissue [4,5,6]. Bacterial infections are the most common causes of sepsis. Approximately half of the patients hospitalized with sepsis have positive cultures [7,8]. In septic patients with positive cultures, Gram-negative bacteria are more prevalent [5,9].

Sepsis can lead to a wide range of complications and organ dysfunction, including but not limited to cardiac dysfunction. Sepsis-induced cardiac dysfunction (SICD) is a type of acute, reversible cardiac dysfunction that occurs in a significant proportion of patients with sepsis [10]. The incidence varies across studies due to differences in cohort sizes and definitions used. A recent meta-analysis reported a 20% occurrence of SICD in patients with sepsis [11]. There are no universally accepted diagnostic criteria for SICD, but it is usually defined as a transient decline in left ventricular ejection fraction (LVEF) of less than 50% and at least a 10% decrease from baseline, which is reversible in 7–10 days [12].

The pathophysiology of SICD has not been fully elucidated, with ongoing research investigating a complex combination of several factors, such as pathogen and damage-associated molecular patterns, inflammation mediators, mitochondrial dysfunction, and calcium metabolism dysregulation, all resulting in myocardial cell injury [13]. In vitro and mouse studies have revealed the molecular mechanisms associated with different bacterial types and their role in causing cardiac dysfunction associated with sepsis [14,15,16,17].

The primary objective of this study was to describe the incidence and outcome implications of SICD in a cohort of culture-positive septic patients in an emergency hospital setting. The secondary purpose was to analyze laboratory markers, especially inflammation and cardiac markers, and microbiological findings in patients with SICD.

## 2. Methods

### 2.1. Study Design and Population

A retrospective, observational analysis was conducted in patients over 18 years old who presented to the Emergency Department and were admitted to the ICU with sepsis as the main diagnosis in an emergency hospital between January 2023 and October 2024. The local ethics committee of Elias University Emergency Hospital approved this study (Protocol number 45/4 January 2023). Sepsis-3 criteria were used to define sepsis and septic shock [1]. All patients with positive cultures who also had laboratory markers and transthoracic echocardiography performed within 24 h of admission were included in the analysis. The exclusion criteria were infective endocarditis, acute coronary syndrome, Takotsubo syndrome, history of heart failure with mildly reduced or reduced ejection fraction, history of cardiomyopathy, history of severe valve disease, history of inflammatory disease, end stage organ disease (liver, kidney, lung), advanced or end-stage oncological or hematological disease.

### 2.2. Definitions and Data Collection

The sepsis diagnosis was characterized by clinical, laboratory, microbiological, and imaging data and categorized based on the source origin of infection (community-acquired or healthcare-associated infections), the primary site of infection, and the causative microorganism. All cultures were collected upon admission. The source of infection was determined based on medical history, symptoms, and clinical examinations. Relevant cultures were collected upon admission in all patients. The types of cultures analyzed included blood, respiratory, urine, intra-abdominal, skin or soft tissue, and bone or joint samples, and they were collected according to the suspected sepsis origin. We collected demographic data (age, gender), data about comorbidities and associated conditions (lung disease, chronic kidney disease, chronic liver disease), and hematological and biochemical markers upon admission (leukocyte count, neutrophil–lymphocyte ratio (NLR), neutrophil count, lymphocyte count, platelet count, C-reactive protein (CRP), procalcitonin, alanine transaminase (ALT), aspartate transaminase (AST), total bilirubin, lactate, troponin, NT-proBNP).

Cardiac function was assessed on transthoracic echocardiography. All exams were performed by experienced cardiologists. Left ventricular systolic dysfunction was considered in patients with a new-onset LVEF less than or equal to 45% and a decrease of at least 10% from baseline, with or without right ventricular systolic dysfunction. LVEF was assessed using the biplane Simpson method whenever possible. In patients with poor acoustic window, the LVEF was visually estimated. Right ventricular systolic dysfunction was considered in patients with a tricuspid annular systolic excursion (TAPSE) of under 17 mm. In patients with poor acoustic window, the right ventricular function was visually estimated. In surviving patients with cardiac dysfunction, follow-up TTEs were reviewed. SICD was confirmed if the systolic dysfunction was reversible within 7–10 days.

### 2.3. Outcomes

The following outcomes were defined: in-hospital mortality, prolonged hospital stay (over 28 days hospitalization), and prolonged ICU stay (over 7-day hospitalization in the ICU).

### 2.4. Statistical Analysis

Statistical analyses were performed using IBM SPSS Statistics for Windows, version 26.0 (IBM Corp., Armonk, NY, USA). The distributions of continuous variables were assessed via the Shapiro–Wilk test. Variables with a normal distribution are reported as the means with standard deviations (SD) and non-normally distributed variables are reported as medians with interquartile ranges (IQR). Categorical variables are presented as frequencies and percentages. Comparisons between groups for normally distributed continuous variables were conducted via independent sample *t* tests. For non-normally distributed data, the Mann-Whitney U test was used. The chi-square test was used to compare categorical variables (or Fisher’s exact test, when frequencies were less than 5). A *p*-value of <0.05 was considered statistically significant. To identify risk factors associated with developing SICD, univariate logistic regression analyses were performed. Odds ratios (OR) with 95% confidence intervals (CI) were reported, and a *p*-value < 0.05 was considered statistically significant.

## 3. Results

**Figure 1:**
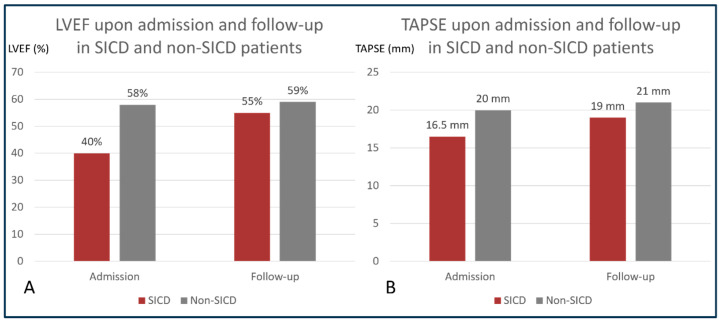
Progression of left and right ventricular function in SICD and non-SICD patients. Panel (**A**): comparison between LVEF upon admission and LVEF at follow-up in patients with and without SICD; (**B**): comparison between TAPSE upon admission and TAPSE at follow-up in patients with and without SICD. LVEF = left ventricular ejection fraction; SICD = sepsis-induced cardiac dysfunction; TAPSE = tricuspid annular systolic excursion.

A total of 100 patients with positive cultures were identified, with a median age 73 (IQR 64, 81), and 55% of them were male. All patients underwent transthoracic echocardiography within 24 h of admission (Table 1). SICD was diagnosed in 14% of patients, with a median age of 73 years (IQR 54, 82), and 71% of them were male. In SICD patients, the median LVEF improved from 40% (IQR 30, 44) upon admission to 58% (IQR 42.5, 63) at 7–10 days follow-up, and the median TAPSE improved from 16.5 mm (IQR 14, 19) to 19 mm (17, 20.5) (Figure 1).

Patients with SICD were more likely to develop septic shock than patients without SICD. Compared with non-SICD patients, SICD patients had slightly longer hospital and intensive care unit stays (Table 1). The in-hospital mortality rate in this study cohort was 44%, and it did not differ between SICD patients and non-SICD patients.

The respiratory tract was the most prevalent infection site leading to sepsis (37%), followed by the urinary tract (29%), abdominal (14%), wound and soft tissue (12%), catheter-related (5%), bone and joint (1%), and ear, nose, and throat (1%).

**Figure 2:**
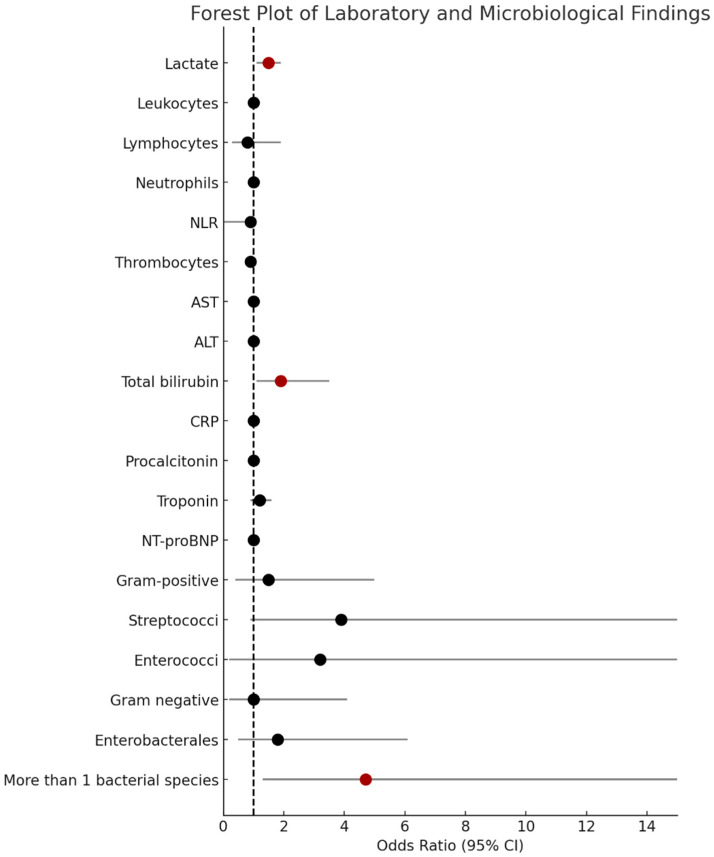
Forest plot of laboratory and microbiological findings associated with SICD. This forest plot illustrates the odds ratios (OR) and the corresponding 95% confidence intervals (CI) for various laboratory and microbiological variables associated with the development of SICD. Each marker represents an individual OR, with statistically significant associations (*p* < 0.05) shown in dark red. ALT = alanine transaminase; AST = aspartate transaminase; NLR = neutrophil–lymphocyte ratio; CRP = C-reactive protein; SICD = sepsis-induced cardiac dysfunction.

Laboratory markers upon hospital admission revealed that SICD patients had significantly higher lactate, AST, and ALT levels upon admission (Table 2). Lactate levels upon admission were a predictive marker for developing SICD even after adjusting for age and sex (OR 1.5, 95% CI: 1.2, 1.96; *p* = 0.001) (Figure 2). “Cardiac” biomarkers, troponin, and NT-proBNP had higher values upon admission in SICD patients but the difference did not reach statistical significance. Similarly, inflammation markers (leukocytes, neutrophils, NLR, CRP, and procalcitonin) were more elevated in patients with SICD than in those without SICD, but the difference did not reach statistical significance (Table 2).

A total of 80% of patients had blood cultures drawn, and 37% were positive. In patients with negative blood cultures, microbiological diagnosis was established based on respiratory cultures (26%), urinary cultures (24%), skin and soft tissue cultures (9%), abdominal surgical intervention (3%), and stool culture (1%). Polymicrobial cultures were identified in five (35%) SICD patients and in nine (10%) non-SICD patients (*p* = 0.025) and were a risk factor for developing SICD (OR 4.7, 95% CI: 1.3,17; *p* = 0.018) (Supplementary File). Patients with SICD had significantly higher rates of streptococcal infections: *Streptococcus* spp. positive cultures were obtained from 29% of the patients with SICD versus 9% of the patients without SICD (*p* = 0.040). The distribution of streptococcal infections in SICD patients were as follows: two patients had *Streptococcus pneumoniae*; one patient had *Streptococcus viridans*; and one patient had *Streptococcus pyogenes*. Streptococcal infections were associated with higher risk of developing SICD (OR 3.9, 95% CI 1–15) and a value of *p* = 0.051 suggesting a trend towards statistical significance. Furthermore, patients with SICD had higher rates of infections with *Enterobacterales*, which was mainly explained by higher rates of *Escherichia coli (E. coli)* and *Proteus mirabilis*, but the difference did not reach statistical significance (Table 2).

There were no patients in the SICD group with positive *Staphylococcus* spp. cultures (either methicillin-sensitive *Staphylococcus aureus*—MSSA, or methicillin-resistant *Staphylococcus* aureus—MRSA).

## 4. Discussion

The aim of this retrospective analysis was to identify patients with cardiac dysfunction associated with sepsis and to discuss their outcomes, laboratory, and microbiological findings.

Age did not differ between the two groups, and the majority of patients who developed SICD were male (71%). Previous reports showed that males seem to be more susceptible to bacterial infection and that there is also a male predominance in patients who develop SICD [18,19].

Mortality was significant—44% of patients during their hospital stay—but it did not differ between SICD and non-SICD patients. Previous studies have shown variable results regarding the prognostic implications for patients who develop SICD. Many indicated that patients who develop SICD have worse outcomes [11]. In this case, the similar mortality rates between patients with SICD vs. without SICD can be explained by the limited sample size but also by the significant mortality rates in sepsis, even in the absence of cardiac dysfunction. Poor outcomes in sepsis have been documented in previous studies, with in-hospital mortality rates of almost 42% in patients with sepsis treated in the ICU [3]. In the current study, patients who developed cardiac dysfunction had longer hospital and ICU stays and were more likely to develop septic shock, which suggests a more severe disease course. An important proportion of the study group developed septic shock (68%). Patients with cardiac dysfunction had higher rates of septic shock (93%), which suggests increased infection severity and higher grade of multiorgan dysfunction.

Patients with SICD had significantly higher lactate levels upon admission than the non-SICD patients, and lactate was found to be a significant predictor of SICD, similar to earlier studies [18,20,21]. Elevated lactate was associated with higher rates of organ dysfunction and worse outcome in septic patients, and measuring lactate levels upon hospital admission is currently recommended in all septic patients [1,22].

Previous studies proposed that SICD patients presented higher inflammation levels. In a study by Sato et al., C-reactive protein levels upon admission were higher in SID patients [20]. In the present study, even though it did not reach statistical significance, SICD patients had clearly higher leukocytes, neutrophils, NLR, CRP, and procalcitonin levels (Table 2). Infection leading to higher inflammatory response and multiple inflammation system activation can lead to cardiac dysfunction by multiple mechanisms. Bacterial infections induce an exaggerated activation of multiple inflammatory pathways which may contribute to myocardial injury and cardiac dysfunction.

Cardiac biomarkers, troponin and NTproBNP, have been analyzed in multiple sepsis cohorts. Even though troponin has an essential and well-established role in diagnosing acute myocardial infarction, positive circulating levels have been reported in other clinical instances. Positive troponin levels in SICD are not considered diagnosis criteria, as cardiac dysfunction can occur even in the absence of troponin elevation. However, measuring troponin upon hospital admission or in the first 24 h does give prognostic information in septic patients and mildly elevated values suggestive of myocardial injury raises the suspicion of cardiac dysfunction [20,23,24,25]. Although NT-proBNP is a key maker in diagnosing heart failure, high values have also been described in septic patients, triggered by myocardial stretch, and have been associated with short- and long-term outcome [20,26]. Septic patients who developed systolic dysfunction had significantly elevated natriuretic peptide levels [20,23,27].

Even though the differences did not reach statistical significance in this study, significant observation can still be noted. There is a clear trend: patients who develop SICD tend to have higher routine laboratory inflammation markers, slightly elevated troponin, and high NT-proBNP levels upon admission. These findings might help identify patients who would benefit from early cardiac imaging.

In a retrospective septic patient cohort analysis, Hanumanthu et al. described culture positivity as an independent risk factor for developing SICD [28]. Based on the assumption that SICD rates might vary depending on the bacterial etiology of sepsis, this study explored potential associations between specific microorganisms and cardiac dysfunction. The current analysis reveals that patients who developed SICD had higher rates of streptococcal and *Enterobacterales* infections. Likewise, patients with cardiac dysfunction had higher rates of positive cultures with more than one bacterial species.

Overall, data on SICD in streptococcal sepsis patients are limited. One important thing to consider in patients with group A streptococcal infections is the possibility of them developing streptococcal toxic shock syndrome. In STSS, bacterial toxins act as “superantigens”, which leads to massive inflammation through excessive T cell activation and cytokine release, resulting in tissue and vascular damage, clinical shock, and organ dysfunction [29]. Streptolysin O seems to be the major streptococcal toxin responsible for cardiac dysfunction in streptococcal infections [17]. In a study comprising 13 patients with *Streptococcus pyogenes* respiratory and skin/soft tissue infections, 10 patients developed cardiac dysfunction [30]. Alhamdi et al. reported that circulating pneumolysin, a virulence factor expressed by *Streptococcus pneumoninae*, induces cardiac contractile depression in cultured cardiomyocytes and leads to elevated troponin levels in mice [31]. In the current study, patients who developed SICD had significantly higher rates of streptococcal infections than patients without SICD (29% versus 9%, respectively).

Gram-negative bacteria are significant causes of sepsis, often leading to severe infections, difficult management due to antibiotic resistance, and contributing overall to poor outcomes. Endotoxins are Gram-negative bacteria membrane components released upon bacterial lysis. They were shown to exhibit a depressor effect on the cardiovascular system and ventricular contractility in several in vitro and in vivo studies [15,32]. Suffredini et al. showed that administrating endotoxins in healthy subjects leads to impaired LV systolic function [33]. There were few cases of reported cardiac dysfunction induced by sepsis in patients with E. coli sepsis [34,35]. In our study, the proportion of Gram-negative bacteria was similar in SICD and non-SICD patients (79% vs. 78%, respectively) but patients with SICD had higher incidence of *Enterobacterales* infections (71% vs. 58%, respectively), with higher incidence of *E. coli* and *Proteus mirabilis*.

Interestingly, no patient with SICD had positive *Staphylococcus* spp. cultures (neither MRSA nor MSSA), which highlights the pathogen-specific mechanisms implicated in developing cardiac dysfunction. Even though animal studies showed the myocardial depressant effect of *Staphylococcus aureus*, there is currently no clearly established association between *Staphylococcus* infections and SICD in humans [36,37,38]. Furthermore, patients who developed cardiac dysfunction had higher rates of positive cultures with more than one microorganism, which may be attributed to higher virulence, multiple pathogenic mechanisms, and higher inflammatory response. Research regarding the association between patients who develop SICD and specific bacterial species remains scarce. Further prospective studies are needed to clarify microbiological-specific mechanisms and their contribution to developing cardiac dysfunction in septic patients.

## 5. Conclusions

In the present study, LV or biventricular systolic dysfunction associated with sepsis occurred in 14% of patients. Although overall mortality did not differ between SICD and non-SICD groups, patients who developed cardiac dysfunction tended to have higher rates of septic shock, and longer hospital and ICU stays, contributing to important long-term outcome implications. Patients who developed SICD had higher lactate, transaminase, inflammation, and cardiac markers levels upon hospital admission, with lactate emerging as a predictive marker even after adjusting for age and sex. The novelty of the study consists of the conjoined rise in inflammatory and cardiac biomarkers, alongside higher lactate, transaminase, and bilirubin levels. These findings point out that this profile of septic patients might be more at risk for developing cardiac involvement and early cardiac imaging should be considered in these cases. Furthermore, SICD patients had significantly higher rates of streptococcal infections and polymicrobial cultures, highlighting possible specific mechanisms implicated in the development of systolic dysfunction during sepsis.

### Limitations

This study has several limitations. The relatively small sample size and retrospective nature of the analysis limit the generalizability of these findings. Obtaining good quality echographic images in critically ill patients can be difficult. The acoustic windows are operator-dependent and can be influenced by several factors, such as patient positioning, and invasive and non-invasive ventilation. Microbiological data from previous hospitalizations in other institutions were not always available, limiting the assessment of the virulence of microorganisms involved in healthcare-associated infections.

## Figures and Tables

**Table 1:** Baseline characteristics. Statistically significant values are shown in bold. Underlined are the 2 wider categories of variables. ICU = intensive care unit; IQR = interquartile range; LVEF = left ventricular ejection fraction; OTI = orotracheal intubation; TAPSE = Tricuspid Annular Plane Systolic Excursion; SICD = sepsis-induced cardiac dysfunction; SOFA = The Sequential Organ Failure Assessment Score.

	SICD (*n* = 14)	Non-SICD (*n* = 86)	*p*
Age, median (IQR)	73 (54, 82)	72.5 (65, 81)	0.371
Male, number (%)	10 (71)	45 (52)	0.183
In-hospital mortality, number (%)	6 (43)	38 (44)	0.926
Hospitalization, days, median (IQR)	14 (7, 24)	10 (7, 19)	0.468
ICU stay, days, median (IQR)	3.5 (1, 12)	2 (1, 6)	0.176
OTI upon admission, number (%)	4 (29)	18 (21)	0.502
Septic shock, number (%)	13 (93)	55 (64)	**0.033**
SOFA Score, median (IQR)	7 (5, 8)	7 (5, 9)	0.678
Healthcare-associated infection, number (%)	4 (29)	39 (45)	0.240
LVEF upon admission, median (IQR)	40 (30, 44)	55 (54, 60)	**<0.001**
TAPSE upon admission, median (IQR)	16.5 (14, 19)	20 (18, 22)	**<0.001**
Comorbidities			
Diabetes mellitus, number (%)	3 (21)	36 (42)	0.146
Obstructive lung diseases, number (%)	0 (0)	16 (19)	0.117
Chronic kidney disease, number (%)	2 (14)	22 (26)	0.508
Chronic hepatitis, number (%)	2 (14)	6 (7)	0.311
Obesity, number (%)	2 (14)	12 (14)	1
Atrial fibrillation, number (%)	7 (50)	35 (40)	0.567
Coronary artery disease, number (%)	0 (0)	7 (8)	0.268
Hypertension, number (%)	11 (78)	77 (89)	0.240
Infection site			
Respiratory, number (%)	3 (21)	34 (39)	0.193
Urinary, number (%)	4 (29)	25 (29)	1
Abdominal, number (%)	1 (7)	13 (15)	0.685
Skin and soft tissue, number (%)	2 (14)	10 (12)	0.674
Catheter-related, number (%)	1 (7)	4 (5)	0.537

**Table 2:** Microbiological and laboratory findings.

		SICD	Non-SICD	*p* Value
Microbiology findings				
Positive blood cultures, number (%)		6 (43)	31 (36)	0.625
Gram-positive bacteria, number (%)		5 (36)	23 (27)	0.527
Staphylococci (MRSA, MSSA), number (%)		0 (0)	13 (15)	0.205
Streptococci, number (%)		4 (29)	8 (9)	**0.040**
Enterococci, number (%)		1 (7)	2 (2)	0.367
Gram-negative bacteria, number (%)		11 (79)	67 (78)	1
*Enterobacterales*, number (%)		10 (71)	50 (58)	0.347
*Escherichia coli*, number (%)		6 (43)	23 (27)	0.222
*Klebsiella pneumoniae*, number (%)		3 (21)	19 (22)	1
*Proteus mirabilis*, number (%)		2 (14)	4 (5)	0.197
*Pseudomonas aeruginosa*, number (%)		0 (0)	9 (10)	0.352
Polymicrobial cultures, number (%)		5 (36%)	9 (10%)	**0.025**
**Laboratory findings upon admission**	**URL**			
Lactate (mmol/L), median (IQR)	1.4 mmol/L	4.5 (3.4, 7.3)	2.3 (1.8, 3.5)	**<0.001**
Leucocytes (10^3^/μL), median (IQR)	10.2 × 10^3^/μL	16.8 (11, 22)	15 (11, 25)	0.980
Lymphocytes (10^3^/μL), median (IQR)	3.4 × 10^3^/μL	0.7 (0.5, 1)	0.9 (0.5, 1.3)	0.548
Neutrophils (10^3^/μL), median (IQR)	6.9 × 10^3^/μL	15 (8.8, 21.5)	13.9 (9.9, 22)	0.972
NLR, median (IQR)		20.4 (12, 31)	16.5 (10.4, 24.4)	0.427
Thrombocytes (10^3^/μL, median (IQR)	400 × 10^3^/μL	211 (140, 240)	226 (142, 293)	0.353
CRP (mg/mL), median (IQR)	10 mg/mL	254 (90, 301)	211 (81, 326)	0.554
Procalcitonin (mg/mL), median (IQR)	0.077 mg/mL	14.6 (3.1, 49)	8.6 (3.4, 33.6)	0.585
AST (U/L), median (IQR)	34 U/L	62 (35, 88)	33.5 (22, 66)	**0.036**
ALT (U/L), median (IQR)	55 U/L	52.5 (32, 70)	35 (22, 58)	**0.048**
Total bilirubin (mg/dL), median (IQR)	1.2 mg/dL	1.2 (0.9, 2.9)	1 (0.7, 1.6)	0.050
Troponin (ng/mL), median (IQR)	0.12 ng/mL	0.68 (0.0, 2.7)	0.1 (0.0, 0.29)	0.097
NT pro BNP (pg/mL), median (IQR)	125 pg/mL	9185 (3100, 13,000)	7270 (2758, 20,400)	0.806

Statistically significant values are shown in bold. Underlined are the 2 wider categories of variables. ALT = alanine transaminase; AST = aspartate transaminase; CRP = C-reactive protein; IQR = interquartile range; MSSA = methicillin-sensitive *Staphylococcus aureus*; MRSA = methicillin-resistant *Staphylococcus aureus*; NLR = neutrophil–lymphocyte ratio; SICD = sepsis-induced cardiac dysfunction; URL = upper reference limit.

## Data Availability

The data that support the findings of this study are available from the corresponding author upon reasonable request.

## Supplementary Materials

The following supporting information can be downloaded at: https://www.mdpi.com/article/10.3390/medicina61101765/s1, Supplementary File: Microbiology findings in patients with polymicrobial cultures.
