# Supplementary material for: Laboratory and Microbiological Considerations in Sepsis-Induced Cardiac Dysfunction
Source: Medicina (Kaunas). 2025 Sep 30;61(10):1765. doi: 10.3390/medicina61101765 (PMC12566532; doi:10.3390/medicina61101765)
Supplement: Supplementary file 1 [file medicina-61-01765-s001.zip › medicina-3879306-supplementary.pdf]

Supplementary File: Microbiology findings in patients with polymicrobial cultures
E. coli= Escherichia coli, MSSA=Methicillin-sensitive Staphylococcus aureus, SICD=sepsis-induced cardiac dysfunction

	**SICD**	**Culture site**	**Result 1**	**Result 2**
**Patient 1**	No	Respiratory	*Acinetobacter baumannii*	*Stenotrophomonas maltophilia*
**Patient 2**	Yes	Skin and soft tissue	*Escherichia coli*	*Streptococcus viridans*
**Patient 3**	No	Blood cultures and respiratory	*MSSA*	*Stenotrophomonas maltophilia*
**Patient 4**	No	Respiratory	*Klebsiella pneumoniae*	*MSSA*
**Patient 5**	Yes	Blood cultures	*Proteus mirabilis*	*Enterococcus faecalis*
**Patient 6**	No	Respiratory	*Klebsiella pneumoniae*	*Acinetobacter baumanii*
**Patient 7**	Yes	Urinary	*Escherichia coli*	*Proteus mirabilis*
**Patient 8**	Yes	Urinary	*Klebsiella pneumoniae*	*Morganella morganii*
**Patient 9**	Yes	Blood cultures and urinary	*Klebsiella pneumoniae*	*Proteus mirabilis*
**Patient 10**	No	Skin and soft tissue	*Pseudomonas aeruginosa*	*Morganella morganii, Serratia marcescens*
**Patient 11**	No	Blood cultures and urinary	*Escherichia coli*	*Klebsiella pneumoniae*
**Patient 12**	No	Respiratory	*MSSA*	*Haemophilus influenzae*
**Patient 13**	No	Skin and soft tissue	*Escherichia coli*	*Proteus mirabilis*
**Patient 14**	No	Respiratory	*MSSA*	*Streptococcus agalactiae*
